# Supplementary material for: Stacking Model‐Based Classifiers for Dealing With Multiple Sets of Noisy Labels
Source: Biom J. 2025 Mar 12;67(2):e70042. doi: 10.1002/bimj.70042 (PMC11898607; doi:10.1002/bimj.70042)
Supplement: Supplementary file 1 — Supporting Information [file BIMJ-67-e70042-s001.zip › density-based-ensemble-model-main/competitors_real_data/netlab-master/netlab-master/help/demopt1.htm]

Netlab Reference Manual demopt1


# demopt1

## Purpose

Demonstrate different optimisers on Rosenbrock's function.

## Synopsis

```
demopt1
demopt1(xinit)
```

## Description

The four general optimisers (quasi-Newton, conjugate gradients,
scaled conjugate gradients, and gradient descent) are applied to
the minimisation of Rosenbrock's well known `banana' function.
Each optimiser is run for at most 100 cycles, and a stopping
criterion of 1.0e-4 is used for both position and function value.
At the end, the trajectory of each algorithm is shown on a contour
plot of the function.

`demopt1(xinit)` allows the user to specify a row vector with
two columns as the starting point. The default is the point [-1 1].
Note that the contour plot has an x range of [-1.5, 1.5] and a y
range of [-0.5, 2.1], so it is best to choose a starting point in the
same region.

## See Also

`conjgrad`, `graddesc`, `quasinew`, `scg`, `rosen`, `rosegrad`

---

**Pages:**
Index


---

Copyright (c) Ian T Nabney (1996-9)
